# Skin Manifestations in COVID-19 Patients: Are They Indicators for Disease Severity? A Systematic Review

**DOI:** 10.3389/fmed.2021.634208

**Published:** 2021-02-16

**Authors:** Parnian Jamshidi, Bahareh Hajikhani, Mehdi Mirsaeidi, Hassan Vahidnezhad, Masoud Dadashi, Mohammad Javad Nasiri

**Affiliations:** ^1^Student Research Committee, School of Medicine, Shahid Beheshti University of Medical Sciences, Tehran, Iran; ^2^Department of Microbiology, School of Medicine, Shahid Beheshti University of Medical Sciences, Tehran, Iran; ^3^Division of Pulmonary and Critical Care, University of Miami, Miami, FL, United States; ^4^Department of Dermatology and Cutaneous Biology, Sidney Kimmel Medical College, Thomas Jefferson University, Philadelphia, PA, United States; ^5^Department of Microbiology, School of Medicine, Alborz University of Medical Sciences, Karaj, Iran

**Keywords:** COVID-19, coronavirus – COVID-19, skin manifestations, skin - pathology, systematic literature search, disease severity, mortality, prognosis

## Abstract

**Introduction:** Until now, there are several reports on cutaneous manifestations in COVID-19 patients. However, the link between skin manifestations and the severity of the disease remains debatable. We conducted a systematic review to evaluate the temporal relationship between different types of skin lesions and the severity of COVID-19.

**Methods:** A systematic search was conducted for relevant studies published between January and July 2020 using Pubmed/Medline, Embase, and Web of knowledge. The following keywords were used: “SARS-CoV-2” or “COVID-19” or “new coronavirus” or “Wuhan Coronavirus” or “coronavirus disease 2019” and “skin disease” or “skin manifestation” or “cutaneous manifestation.”

**Results:** Out of 381 articles, 47 meet the inclusion criteria and a total of 1,847 patients with confirmed COVID-19 were examined. The overall frequency of cutaneous manifestations in COVID-19 patients was 5.95%. The maculopapular rash was the main reported skin involvement (37.3%) commonly occurred in middle-aged females with intermediate severity of the disease. Forty-eight percentage of the patients had a mild, 32% a moderate, and 20% a severe COVID-19 disease. The mild disease was mainly correlated with chilblain-like and urticaria-like lesions and patients with vascular lesions experienced a more severe disease. Seventy-two percentage of patients with chilblain-like lesions improved without any medication. The overall mortality rate was 4.5%. Patients with vascular lesions had the highest mortality rate (18.2%) and patients with urticaria-like lesions had the lowest mortality rate (2.2%).

**Conclusion:** The mere occurrence of skin manifestations in COVID-19 patients is not an indicator for the disease severity, and it highly depends on the type of skin lesions. Chilblain-like and vascular lesions are the ends of a spectrum in which from chilblain-like to vascular lesions, the severity of the disease increases, and the patient's prognosis worsens. Those with vascular lesions should also be considered as high-priority patients for further medical care.

## Introduction

A viral outbreak caused by severe acute respiratory syndrome coronavirus 2 (SARS-CoV-2) emerged from Wuhan, China in late December 2019 (1). The disease was named coronavirus disease 2019 (COVID-19) by World Health Organization (WHO) and was declared as a pandemic on 11 March 2020 (2). After 1 year from the beginning of the pandemic, the full spectrum of COVID-19 presentations and its relationship with disease severity is still unknown. Fever, cough, chills, dyspnea, myalgia, and sore throat are the most common clinical presentations of COVID-19 and as time goes on, different other manifestations have been reported (3). Recently, skin lesions have been described as potential manifestations of COVID-19 (4–6). The cutaneous changes reported to date include maculopapular rash, vesicular lesions, urticaria-like lesions, and chilblain-like lesions (4–8). Some of these skin manifestations arise before the signs and symptoms more commonly associated with COVID-19, suggesting that they could be presenting signs of COVID-19 (9). However, the link between skin manifestations and the severity of the disease remains debatable. Due to the great variety of reported dermatologic presentations as well as the inconsistency of data on the association between skin presentations of COVID-19 with poor outcome, we aimed to conduct a comprehensive systematic review on the clinical and histopathological characteristics of skin manifestations in relation to other features of confirmed COVID-19 patients and to evaluate the temporal relationship between different types of skin lesions and the severity of COVID-19.

## Methods

This review conforms to the “Preferred Reporting Items for Systematic Reviews and Meta-Analyses” (PRISMA) statement (10). Registration: PROSPERO (pending registration ID: 215422).

### Search Strategy and Selection Criteria

To investigate the prevalence and characteristics of cutaneous manifestations in COVID-19 patients, a systematic search was conducted for relevant studies published between January and July 2020 using Pubmed, Embase, and Web of knowledge.

The following search terms were used (designed using MeSH keywords and Emtree terms): “SARS-CoV-2” or “COVID-19” or “new coronavirus” or “Wuhan Coronavirus” or “coronavirus disease 2019” and “skin disease” or “skin manifestation” or “cutaneous manifestation.” Only studies included if they contained data about the skin manifestation in patients with confirmed COVID-19. There were no language restrictions. We got help from the Google Translate system for non-English papers. Review articles, duplicate publications, and articles with no relevant data were excluded from the analysis. Two authors independently screened the remaining articles. Finally, selected data were extracted from the full-texts of eligible publication by other investigators of the team.

#### Data Extraction

Data about the first author's name, date of publication, country, number of COVID-19 patients, number of cases with skin manifestations, age, gender, location and type of skin manifestations, associated cutaneous symptoms, the onset of skin lesions with systemic symptoms, the median duration of the lesions, treatment strategies and main histological findings of the lesions as well as comorbidities, associated symptoms, drug history, laboratory findings, severity and outcome of the patients were selected for further analysis. All cutaneous presentations related to COVID-19 were categorized into six groups: chilblain-like, vesicular, urticaria-like, maculopapular, vascular, and miscellaneous (lesions that we couldn't subscribe to any of the groups). Petechiae, purpura, livedo, and necrosis were classified into vascular lesions. Two authors (PJ, BH) independently extracted the data from the selected studies. The data was jointly reconciled, and disagreements were discussed and resolved between review authors (PJ, BH, MJN).

#### Quality Assessment

The critical appraisal checklist for case reports provided by the Joanna Briggs Institute (JBI) was used to perform a quality assessment of the studies (11).

## Results

**Figure 1:**
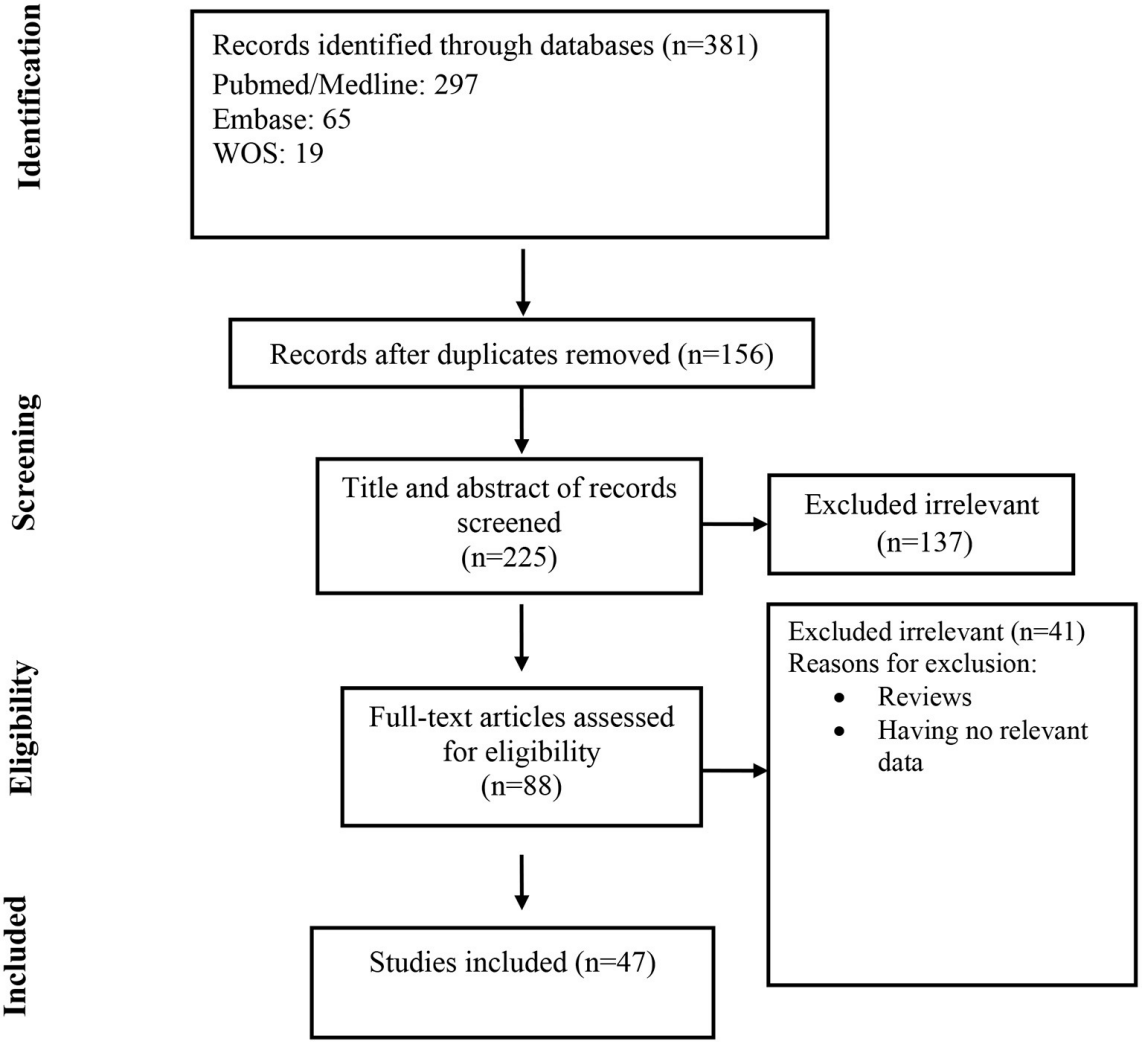
Flow chart of study selection for inclusion in the systematic review and meta-analysis.

At the first round of review, 381 articles were selected. After removing the duplicates and studies that did not meet the entry criteria, 88 full texts were finally selected for further assessment. Of these, only 47 articles had the characteristics appropriated for systematic review and were entered into the data extraction (Figure 1). Most of the studies were case reports (47%, N: 22) followed by case series (42.4%, N: 20), retrospective hospital/private section-based study (6.4%, N: 3), and cross-sectional (4.2%, N: 2). Thirteen articles were originated from Italy, 11 from Spain, 10 from France, 5 from the USA, and others from Belgium, China, Thailand, Kuwait, Indonesia, Russia, Turkey, and Singapore. Information of the 47 analyzed articles can be found in Table 1.

**Table 1:** Characteristics of the included studies.

**First author**	**Country**	**Published time**	**Type of study**	**Mean age**	**Male/ Female**	**No. of confirmed COVID-19 patient(s)**	**No. of the patient(s) with skin manifestations**
Hunt, M. (12)	USA	Mar 28-2020	Case report	20	1 M	1	1
^*^Recalcati, S. (6)	Italy	Apr 7-2020	Retrospective hospital-based study	–	–	88	18
^*^Joob, B. (13)	Thailand	Apr 7-2020	Retrospective hospital-based study	40.5	4 M, 37 F	41	1
Zhang, Y. (14)	China	Apr 7-2020	Case series	59	4 M, 3 F	7	7
Fiehn, C. (15)	Spain	Apr 15-2020	Case report	28	1 F	1	1
Mahé, A. (16)	France	Apr 16-2020	Case report	46	1 F	1	1
Zulfiqar, A. A. (17)	France	Apr 16-2020	Case report	65	1 F	1	1
^*^Magro, C. (18)	USA	Apr 18-2020	Case series	54.6	3 M, 2 F	5	3
Morey-Olive, M. (19)	Spain	Apr 22-2020	Case series	Boy: 6 years Girl: 2 months	1 M, 1 F	2	2
Najarian, D. J. (20)	USA	Apr 22-2020	Case report	58	1 M	1	1
Gianotti, R. (21)	Italy	Apr 29-2020	Case series	68	1 M, 2 F	3	3
Gianotti, R. (22)	Italy	Apr 30-2020	Case series	–	–	5^**^	5^**^
Sanchez, A. (23)	France	Apr 30-2020	Case report	Elderly	–	1	1
Galván Casas, C. (24)	Spain	Apr 30-2020	Cross-sectional	56.3	113 M, 121 F	234	234
Ahouach, B. (25)	France	May 1-2020	Case report	57	1 F	1	1
Quintana-Castanedo, L. (26)	Spain	May 1-2020	Case report	61	1 M	1	1
Marzano, A. V. (27)	Italy	May 1-2020	Case series	56.4	16 M, 6 F	22	22
Zengarini, C. (28)	Italy	May 3-2020	Case report	67	1 F	1	1
Alramthan, A. (29)	Kuwait	May 5-2020	Case series	31	2 F	2	2
Henry, D. (30)	France	May 5-2020	Case report	27	1 F	1	1
^*^Recalcati, S. (31)	Italy	May 6-2020	Case series	72.2	58 M, 49 F	107	3
^*^Tammaro, A. (32)	Italy	May 6-2020	Case series	–	–	130 + X^***^	2 + 1^***^
van Damme, C. (33)	Belgium	May 6-2020	Case report	71	1 M	1	1
Avellana Moreno, R. (34)	Spain	May 6-2020	Case report	32	1 F	1	1
Amatore, F. (35)	France	May 6-2020	Case report	39	1 M	1	1
Suarez-Valle, A. (36)	Spain	May 8-2020	Case series	–	–	3	3
Fernandez-Nieto, D. (37)	Spain	May 8-2020	Case series	45	6 M, 18 F	24	24
Paolino, G. (38)	Italy	May 8-2020	Case report	37	1 F	1	1
Diaz-Guimaraens, B. (7)	Spain	May 8-2020	Case report	48	1 M	1	1
Bouaziz, J. D. (39)	France	May 8-2020	Case series	–	–	14	14
Locatelli, A. G. (40)	Italy	May 9-2020	Case report	16	1 M	1	1
Jimenez-Cauhe, J. (41)	Spain	May 9-2020	Case series	66.7	4 F	4	4
Robustelli Test, E. (42)	Italy	May 10-2020	Case report	70	1 F	1	1
Gunawan, C. (43)	Indonesia	May 10-2020	Case report	51	1 M	1	1
^*^de Masson, A. (44)	France	May 28-2020	Retrospective private practices-based study	–	–	25	7
Freeman, E. E. (45)	USA	May 30-2020	Case series	41	12 M, 11 F	23	23
Bosch-Amate, X. (46)	Spain	June 03-2020	Case report	79	1 F	1	1
Reymundo, A. (47)	Spain	June 04-2020	Case series	66.6	2 M, 5 F	7	7
Gargiulo, L. (48)	Italy	June 07-2020	Case report	72	1 F	1	1
Freeman, E. E. (45)	USA	June 23-2020	Case series	44	78 M, 93 F	165^****^	165^****^
^*^Askin, O. (49)	Turkey	June 24-2020	Cross-sectional	NM	NM	122	34
Ciccarese, G. (50)	Italy	June 24-2020	Case report	19	1 F	1	1
^*^Matar, S. (51)	France	June 26-2020	Case series	55.6	6 M, 2 F	759	8
Ho, W. Y. B. (52)	Singapore	June 26-2020	Case series	59	1 M, 1 F	2	2
Potekaev, N. N. (53)	Russia	July 03-2020	Case series	62.2	7 M, 5 F	12	12
Le Cleach, L. (54)	France	July 06-2020	Case series	34	3 M, 7 F	10	10
Proietti, I. (55)	Italy	July 22-2020	Case report	6 months	1 M	1	1

* *Articles that are included for calculating the prevalence of cutaneous manifestations in confirmed COVID-19 patients*.

** *Total population of cases was 8 but data of 5 patients were available only*.

*** *Tammaro et al. visited 130 patients in a hospital in Rome in which 2 patients had cutaneous manifestations. Also, they visited undetermined (X) patients in Barcelona in which there was a patient with cutaneous manifestation. Note that for calculating the prevalence number we excluded the latter patient (because of the undetermined number of total case population)*.

**** *Total population of confirmed COVID-19 patients was 171 but data of 165 patients were available only*.

A total of 1,847 patients with confirmed COVID-19 (based on positive RT-PCR or positive antibody tests) were examined in 47 articles, of which 597 patients had different skin manifestations. The overall frequency of cutaneous manifestations in COVID-19 patients was 5.95%.

### Characteristics of the Cutaneous Lesions in Confirmed COVID-19 Patients

The maculopapular rash was the main reported skin involvement (37.3%) followed by chilblain-like lesions (18.4%). The prevalence rate of vesicular and urticaria-like lesions was 15% (**Table 4**).

The mean age of patients with cutaneous manifestations was 53.3 (ranging from 16 to 92) years. Chilblain-like lesions were more common in younger patients (mean age: 40.7 years) and vascular lesions were more common in the elderly (mean age: 72.3 years).

The prevalence of skin lesions was slightly higher in females than males (54 vs. 46%). Urticaria-like, chilblain- like and miscellaneous lesions were more frequent among females (**Table 4**). Vascular lesions were more frequent in males (61%). The prevalence of vesicular and maculopapular lesions was almost the same in men and women (51 and 49%).

Trunk, lower limb, and upper limb were the main involved regions. Chilblain-like and vascular lesions were more common in acral areas and except for maculopapular lesions, others were commonly located in the trunk. The maculopapular lesions were more common in extremities. The involvement of palms and soles were rare. Mucous membrane involvement was reported in all types of skin lesions particularly maculopapular and vascular lesions, but it was not reported in chilblain-like lesions (**Table 4**). Vesicular rashes could have diffused polymorphic or localized monomorphic patterns (27, 37).

Out of 597, 397 (66%) of the patients had associated cutaneous symptoms. Pruritus was the most prominent (238, 60%) particularly in vesicular lesions (89%). Pain was the most frequent symptom in chilblain-like lesions (63.5%) (see **Table 4**).

In the majority of patients (89.5%), dermatologic manifestations presented after (55%) or at the same time (34.5%) with the onset of systemic symptoms of COVID-19. Urticaria-like lesions appeared usually as a concomitant symptom (47%). In 3.5% of patients particularly with chilblain-like lesions, skin manifestations were the only presentation of COVID-19. In 7% of patients, skin manifestations occurred before the systemic symptoms, particularly in chilblain-like lesions (**Table 4**).

The median duration of skin lesions was about 9 days ranging from 1 to 18 days (**Table 4**). Urticaria-like lesions had the least duration (5 days) and chilblain-like lesions had the most duration (14 days).

No skin biopsy or histological examination of urticaria-like lesions was performed. Therefore, the following results are related to other types of skin lesions.

Perivascular lymphocytic infiltration, spongiotic and interface dermatitis, and vacuolization or keratinocyte necrosis were the common histologic findings in skin biopsies, except for vesicular lesions. In vesicular lesions, the absence of inflammatory infiltrates, atrophic epidermis, and hyperkeratosis was reported. In almost all types of lesions (except maculopapular and vesicular lesions) thrombotic vasculopathy and red blood cell extravasation were present. Langerhans cell aggregations were seen within the epidermis in maculopapular lesions. Telangiectatic blood vessels were seen within the dermis of vascular and miscellaneous lesions. Virally-induced cytopathic alterations were absent according to reports on the miscellaneous category. Striking vascular and dermal deposits of complement factors (C5b-9, C3d, C4d) and IgM were present in four vascular rashes. Some studies performed an RT-PCR test on skin samples of maculopapular and vesicular lesions and the results were all negative for SARS-CoV-2. More details can be found in Table 2.

**Table 2:** Characteristics of the cutaneous lesions in confirmed COVID-19 patients.

**First author**	**Category**	**Location**	**Description**	**Associated cutaneous symptoms**	**Rash onset with other symptoms**	**Median duration**	**Main histologic findings**	**Rash treatment**
Locatelli, A. G. (40)	Chilblain-like	Dorsal aspects of the fingers	Erythematous-oedematous macules and plaques (Chilblain-like)	Asymptomatic	After	–	Oedema of the papillary dermis, superficial and deep lymphocytic infiltrate in a peri-vascular and strong peri-eccrine pattern; no signs of endothelial damage. consistent with a diagnosis of chilblains	–
Alramthan, A. (29)	Chilblain-like	Dorsal aspect of fingers bilaterally, subungual area of the thumb	Red-purple papules, diffused erythema in the subungual area	–	Chief complaint	–	–	–
Suarez-Valle, A. (36)	Chilblain-like	Toes (3), soles (1) (sparing palms and mucous membranes)	Rounded reddish-purple plaques, measuring between 0.5 and 1 cm, sharply defined, with no retiform borders	–	23 days after	14 days	Ischemic necrosis affecting the epidermis and dermis with signs of re-epithelialization. vasculitis or microthrombi were not found.	–
de Masson, A. (44)	Chilblain-like	Hands and feet	Acral lesions (chilblains)	–	–	–	Lichenoid dermatitis with a perivascular and eccrine mononuclear infiltrate, vascular microthrombi	–
Freeman, E. E. (45)	Chilblain-like	Hand (7), foot (20)	Pernio-like acral skin lesions	Pruritus (8), Pain/Burning (16)	Before (4), After (11), At the same time (3), No other COVID-19 symptoms (5)	14 days	Mild vacuolar interface dermatitis with dense superficial and deep lymphocytic inflammation, consistent with pernio vs. connective tissue disease. No thrombi were noted.	–
Le Cleach, L. (54)	Chilblain-like	Acral area of hand and foot, dorsum of toes and soles, lateral part of the foot	Typical chilblains, severe form with bullae, erythema multiform-like lesions, punctiform purpuric lesions, diffuse vascular erythema, and oedema	–	After/Before	–	Vacuolization or apoptosis of keratinocytes, superficial and deep infiltrates mainly of lymphocytes, perieccrine, and perivascular reinforcement, superficial capillary thrombosis, dermal oedema	Without treatment, topical corticosteroids
Najarian, D. J. (20)	Maculopapular	Legs, thighs, forearms, arms, shoulders, back, chest, and abdomen (sparing the face, hands, feet, and mucosa)	Morbilliform erythematous macules and patches	Pruritus	After	–	–	Triamcinolone 0.1%
Sanchez, A. (23)	Maculopapular	Trunk, back, thighs, arms	Digitatepapulosquamous eruption and erythematous periumbilical patch (digitate scaly thin plaques)	–	7 days after	7 days	Spongiosis, parakeratosis, a few rounded spongiotic vesicles containing aggregates of lymphocytes and Langerhans cells. moderate lymphohistiocytic infiltration, negative COVID-19 RT-PCR on a fresh skin biopsy specimen	No
Hunt, M. (12)	Maculopapular	Trunk and extremities (sparing the face, mucosal or ocular involvement)	Diffuse, morbilliform, maculopapular (consistent with a viral exanthem)	Non-pruritic	At the same time	–	–	–
Ahouach, B. (25)	Maculopapular	Trunk, limbs	Diffuse fixed erythematous blanching maculopapular lesions	Burning	–	–	Spongiosis, basal cell vacuolation and mild perivascular lymphocytic infiltrate, negative PCR on whole-skin biopsy specimen for SARS-CoV-2.	–
AvellanaMoreno, R. (34)	Maculopapular	Face, neck, thorax, abdomen, buttocks, extremities including folds and scalp, respecting the palmoplantar region and mucosa	Generalized, pruritic morbilliform rash cephalocaudal progress (petechial and maculopapular on an erythematous base), a scaly reaction occurred on the fourth day after the rash started	Pruritus	6 day after	4 days	–	–
Reymundo, A. (47)	Maculopapular	Trunk (7), proximal upper limbs (6), proximal lower limbs (1)	–	–	–	–	Mild superficial perivascular lymphocytic infiltrate	Without treatment (1), systemic corticosteroid (6)
Morey-Olive, M. (19)	Maculopapular (1), Urticaria-like (1)	*Maculopapular:* trunk, neck spreading to the cheeks, upper and lower extremities (involving the palms) *Urticaria-like:* face, upper extremities spreading to the trunk and lower extremities (sparing palms and soles)	*Maculopapular:* erythematous, confluent, non-pruritic maculopapular exanthem *Urticaria-like:* pruritic Urticaria-like exanthem	Pruritus (1)	After (1), At the same time (1)	5 days	–	No
Gianotti, R. (22)	Maculopapular (3), Vascular (2)	Trunk, limb	*Maculopapular:*diffusemaculo-papulovesicular rash and hemorrhagic dot-like area, sligthlypapular erythematous exanthema, erythematous papular eruption with crusted and erosive lesions mimicking Grover disease *Vascular:* diffuse macular livedoid hemorrhagic lesions	–	After (5)	–	*Maculopapular:* classic dyskeratotic cells, ballooning multinucleated cells and sparse necrotic keratinocytes with lymphocytic satellitosis, edematous dermis with many eosinophils, lymphocytic vasculitis *Vascular:* diffuse telangiectatic small blood vessels in the dermis, spongiotic dermatitis with exocytosis along with a large nest of Langerhans cells and a dense perivascular lymphocytic and eosinophilic infiltration, lymphocytic vasculitis, Intravascular microthrombi in the small dermal vessels.	–
Mahé, A. (16)	Miscellaneous	Both antecubital fossae extended to the trunk and axillary folds	Erythematous rash	–	4 days after	5 days	–	–
Robustelli Test, E. (42)	Miscellaneous	Face, trunk, upper and lower limbs (sparing the mucous membranes, palms, and soles)	Diffuse pustular eruption: widespread eruption on an erythematous-oedematous base, with scattered pinhead-sized pustules and scales, Targetoid lesions studded with small pustules in a symmetric pattern	Pruritus	After	–	Subcorneal pustule with mild focal acanthosis and spongiosis, neutrophilic exocytosis, sparse keratinocyte necrosis, perivascular lymphocytic infiltrate with rare neutrophils and eosinophils (consistent with AGEP)	–
Zengarini, C. (28)	Miscellaneous	Neck, trunk, back, proximal portions of limbs (sparing the palmoplantar skin, face, and mucous membranes)	Erythematous confluent rash, with undefined margins, bleaching	Moderate pruritus	After	7 days	Slight superficial perivascular lymphocytic infiltrate extremely dilated vessel in the papillary and mid dermis.	–
Amatore, F. (35)	Miscellaneous	Upper limbs, chest, neck, abdomen, and palms (sparing the face and mucous membranes)	Erythematous and edematous annular fixed plaques	Non-pruritic	At the same time	7 days	Predominantly superficial perivascular infiltrate of lymphocytes without eosinophils, papillary dermal edema, subtle epidermal spongiosis, lichenoid, and vacuolar interface dermatitis with occasional dyskeratotic keratinocytes in the basal layer. No virally-induced cytopathic alterations or intranuclear inclusions were present. Direct immunofluorescence was negative.	HCQ
Gargiulo, L. (48)	Miscellaneous	Trunk, upper and lower limbs	Erythema multiforme-like (erythematous and slightly edematous patches, along with some isolated typical target lesions)	Pruritus	10 days Before	–	Mixed perivascular and interstitial infiltrate including lymphocytes, granulocytes, histiocytes, plasma cells, and mast cells.	Systemic corticosteroid
Ciccarese, G. (50)	Miscellaneous	Lower limbs, Inner surface of the lips, platelet, gingiva	*Cutaneous lesions:* erythematous macules, papules, and petechiae *Oropharyngeal lesions:* erosions, ulcerations, blood crusts, petechiae	Asymptomatic	5 days after	12 days	–	–
Recalcati, S. (6)	Miscellaneous (14), Urticaria-like (3), Vesiclular (1)	Trunk	Erythematous rash (14), widespread urticaria (3), chickenpox-like vesicles (1)	Low or absent pruritus	At the same time (8), After (10)	Few days	–	–
Gianotti, R. (21)	Maculopapular (3)	Arms, trunk, lower limbs	Widespread erythematous macules, erythematous crusted macules, and papules	Pruritus (1)	After (2), Before (1)	5, 8, 10 days	Perivascular dermatitis with slight lymphocytic exocytosis in a vasculitic pattern. vascular thrombosis, Swollen thrombosed vessels with neutrophils, eosinophils and nuclear debris, extravasated red blood cells, focal acantholyticsuprabasal clefts, dyskeratotic and ballooning herpes-like keratinocytes, swollen vessels in the dermis with dense lymphocyte infiltration, mixed with rare eosinophils. a nest of Langerhans within the epidermis.	No (3)
Jimenez-Cauhe, J. (41)	Miscellaneous (4)	Upper trunk, coalescing in the back, and then spread to the face and limbs within 1 week (without the involvement of palms and soles), platal macules and petechiae (3)	Erythematous papules that progressively turned to erythemato-violaceous patches with a dusky center, and a pseudo-vesicle in the middle, typical target lesions (2).	–	19.5 days after	17.5 days	Normal basket-weave stratum corneum, mild to moderate spongiosis in the epidermis, dilated vessels filled with neutrophils, extravasation of red blood cells, lymphocytic perivascular and interstitial infiltrate, Basal vacuolar changes with interface dermatitis, lymphocytic exocytosis	Systemic corticosteroids
Galván Casas, C. (24)	Chilblain-like (29), Vesicular (17), Urticaria-like (49), Maculopapular (122), Vascular (17)	*Chilblain-like:* acral areas of hands and feet. usually asymmetrical *Vesicular*: trunk, limbs *Urticaria-like:* mostly trunk, a few cases were palmar *Maculopapular*: extremities, mostly dorsum of the hands *Livedo/necrosis:* trunk, acral area	Pseudo-chilblain (29), Vesicular (17), Urticarial (49), Maculopapules (122), Livedo/necrosis (17)	–	Before (9), At the same time (147), After (77)	*Chilblain-like:* 12.7 days *Vesicular:* 10.4 days *Urticaria-like:* 6.8 days *Maculopapular*: 8.6 days	–	–
Fiehn, C. (15)	Urticaria-like	Both heels	Confluent erythematous-yellowish papules and plaques	Pruritus	13 days After	–	–	–
Gunawan, C. (43)	Urticaria-like	Face	Urticaria	Pruritus	5 days After	1		Loratadine
Quintana-Castanedo, L. (26)	Urticaria-like	Thighs, arms, and forearms (sparing the palms and soles)	Urticarial rash consisting of confluent, edematous, and erythematous papules	Mild pruritus	Chief complaint	7 days	–	Antihistamine
Henry, D. (30)	Urticaria-like	Face, acral area, palm	Disseminated erythematous plaques (Urticaria), papules in palm	Pruritus	Before	–	–	Antihistamine
van Damme, C. (33)	Urticaria-like	–	Extensive acute urticarial rash	–	At the same time	–	–	Bilastine
Paolino, G. (38)	Urticaria-like	Trunk, neck, face, lower limbs	An urticaria-like lesion with craniocaudal development	Non-pruritic	3 days after	8 days	–	–
Proietti, I. (55)	Urticaria-like	Trunk, limbs	Giant urticaria with multiple lesions	–	14 days after RT-PCR test (no associated symptoms)	–	–	Oral betamethasone
Zulfiqar, A. A. (17)	Vascular	Lower extremity	Purpura	–	5 days after	13 days	–	IVIG, Prednisolone, Platelet transfusion
Joob, B. (13)	Vascular	–	Petechiae	–	–	–	–	No
Diaz-Guimaraens, B. (7)	Vascular	Symmetric periflexural distribution: buttocks, popliteal fossae, proximal anterior thighs, and lower abdomen (sparing the crural folds and mucosa)	Confluent erythematous macules, papules, and petechiae	Slightly pruritic	3 days after	5 days	Perivascular lymphocytic infiltrate, red cell extravasation, and focal papillary edema, along with focal parakeratosis and isolated dyskeratotic cells. No features of thrombotic vasculopathy were present	0.05% Betamethasone dipropionate cream, Loratadine
Magro, C. (18)	Vascular	Buttocks, palms and soles, chest, legs, and arms	Purpuric reticulated eruptions with surrounding inflammation (Livedoracemosa)	–	4 days after	–	Thrombogenic vasculopathy, extensive necrosis of the epidermis and adnexal structures, interstitial and perivascular neutrophilia with prominent leukocytoclasia, superficial vascular ectasia, perivascular lymphocytic infiltration, absence of clear vasculitis, Significant vascular deposits of C5b-9, C3d, and C4d (in all cases)	–
Recalcati, S. (6)	Vascular	Acral area, foot	Acrocyanosis (2), foot thrombosis (1)	–	–	–	–	–
Zhang, Y (14)	Vascular	Finger/toe	Acro-ischemia including cyanosis, bulla, and dry gangrene	–	–	12 days to death	–	–
Bosch-Amate, X. (46)	Vascular	Both legs	Retiform purpuric-violaceous patches of 15 cm with some hemorrhagic blisters and crusts suggestive of retiform purpura	Pain	–	–	Multiple thrombi occluding most small-sized vessels of the superficial and mid-dermis, deposition of IgM, C3, C9, and fibrinogen within superficial-to-deep dermal blood vessel walls.	–
Bouaziz, J. D. (39)	Vesicular (2), Urticaria-like (1), Chilblain-like (2), Vascular (3), Miscellaneous (6)		Inflammatory lesions were reported in 7 patients: exanthema (4), chickenpox like vesicles (2), cold urticaria (1), Vascular lesions were reported in 7 patients: violaceous macules with “porcelain-like” appearance (1), livedo (1), nonnecroticpurpura (1), necrotic purpura (1), chilblain appearance with Raynaud's phenomenon (1), chilblain (1), eruptive cherry angioma (1).	–	A few days after	–	–	–
Marzano, A. V. (27)	Vesicular (22)	Trunk (22), limbs (4)	Scattered (16), diffuse (6), Predominance of vesicles (12), varicella-like exanthem	Mild pruritus (9)	3 days after	8 days	Basket-wave hyperkeratosis, absence of inflammatory infiltrate, atrophic epidermis, vacuolar alteration with disorganized keratinocytes lacking orderly maturation, enlarged and multinucleate keratinocytes with dyskeratotic (apoptotic) cells.	–
Fernandez-Nieto, D. (37)	Vesicular (24)	Head (4), anterior trunk (21), posterior trunk (14), arms (8), legs (10), palms-soles (2)	18 disseminated pattern (small papules, vesicles, and pustules with varying sizes of up to 7–8 mm diameter, different stages of the lesions appeared simultaneously), 6 localized pattern (monomorphic lesions, of up to 3–4 mm diameter, at the same stage of evolution, mostly trunk involvement)	Pruritus (20), Asymptomatic (4)	Before (2), At the same time (3), After (19)	10 days	Intraepidermal vesicles with mild acantolisis and ballooned keratinocytes consistent with a viral infection, negative SARS-CoV-2 RT-PCR on fluid content of the vesicles	–
Tammaro, A. (32)	Vesicular	Trunk, back	Isolated herpetiform lesions: lesions were characterized by vesicles surrounded by erythematous halos. In one of the patients, the vesicles had started to form crusts, numerous vesicular isolated lesions on her back.	Mild pruritus	After	–	–	–
Potekaev, N. N. (53)	Chilblain-like (1), Vesicular (2), Urticaria-like (1), Maculopapular (4), Vascular (4)	Lower limb (6), upper limb (5), trunk (4), first MTP joints (1), ankles and dorsal surfaces of the feet and toes (1)	*Vascular:* papulonecrotic rash with hemorrhagic crusts, polymorphic cutaneous vasculitis, dense petechial and ecchymotic rash *Chilblain-like:* hyperemic pernio-like lesions *Maculopapular:* spotted elements of bright pink color, papulosquamous rash (pytriasis rosea-like, absence of the herald patch), disseminated pink-red maculopapular rash resembling that of measles, large bright red foci *Vesicular:* papulovesicular eruptions with surface erosions	Pain (1), Pruritus (1)	Before (1), At the same time (1), After (5)	–	–	Without treatment (1), Systemic corticosteroid (3)
Freeman, E. E. (56)	Chilblain-like (31), Vesicular (18), Urticaria-like (27), Maculopapular (78), Vascular (11)	Hand (38), foot (51), face (32), head (11), neck (26), chest (49), abdomen (63), back (62), arm (66), genitals (7), leg/buttocks (72), entire body (9)	*Maculopapular:* morbilliform rash, macular erythema, papulosquamous *Vascular:* retiform purpura	Pruritus (97), Burning/pain (55)	Before (17), After (107), At the same time (29), Chief complaint (10)	–	*Vascular:* thrombotic vasculopathy, leukocytoclastic vasculitis *Maculopapular:* spongiosis and dermal inflammation *Chilblain-like:* vacuolar interface dermatitis, subepidermal edema, and superficial and deep lymphocytic inflammation *Miscellaneous (actually distributed petechial, macular, and urticarial eruption):* numerous dyskeratotic keratinocytes, sparse perivascular lymphohistiocytic inflammation, and rare dermal eosinophils.	–
Matar, S. (51)	Maculopapular, Vesicular, Miscellaneous	-	Disseminated maculopapular exanthema, digitate papulosquamous rash, herpes recurrence, papulovesicular rash, Grover's disease	–	13 days after	–	–	–
Ho, W. Y. B. (52)	Miscellaneous (1), Vascular (1)	*Miscellaneous:* Trunk, proximal thighs, intertriginous areas including bilateral axillae and groin *Vascular:* abdomen, back	*Miscellaneous:* erythematous, blanchable, non-follicular papules, non-follicular pinpoint pustules within the intertriginous areas *Vascular:* purpuric plaques	–	*Miscellaneous:* 12 days after *Vascular:* 15 days after	7 days, 10 days	*Miscellaneous:* spongiotic and interface dermatitis, superficial perivascular infiltrate of predominantly lymphocytes, focal erythrocyte extravasation without vasculitis. There were no viral cytopathic or herpetic changes.	Topical corticosteroids (2)

Most lesions required systemic corticosteroids (47%) or had spontaneous remission (23.5%). Antihistamines were the most widely used medication especially for urticaria-like lesions (57%). Systemic corticosteroids were commonly used in vascular lesions (71%) (Tables 2, **4**).

### Characteristics of the Confirmed COVID-19 Patients With Skin Manifestations

The overall prevalence of comorbidities among patients with skin manifestations was 17.9% (**Table 4**). Hypertension (39%), diabetes (23%), and dermatologic diseases (20%) were the most frequent comorbidities, respectively. Utmost cases with comorbidity were across the patients with maculopapular lesions (40%). Previous dermatologic illnesses were most common in patients with vesicular lesions (**Table 4**). Cardiovascular disease, hypertension, and obstructive lung diseases were common comorbidities amongst patients with vascular lesions (**Table 4**). Rheumatologic diseases were more frequent in patients with chilblain-like lesions (30%). Diabetes was seen commonly in patients with urticaria-like lesions (46%).

Fever (72%), cough (61%), fatigue/myalgia (51%), and dyspnea (46%) were the most common associated symptoms amongst the patients. Fever was more frequent in patients with vascular lesions (84%) and less frequent in patients with chilblain-like lesions (39.5%). Headache (41%), dysosmia/hyposmia (27.5%), nasal congestion/coryza (19%), and irritability/confusion (10%) were mainly seen in patients with vesicular lesions. Seventeen percentage of patients with chilblain-like lesions, 5% of patients with urticaria-like lesions, and 1% of patients with maculopapular lesions were asymptomatic. Bleeding presentations like epistaxis were seen just in patients with vascular lesions (Tables 3, 4).

**Table 3:** Characteristics of the confirmed COVID-19 patients with skin manifestations.

**First author**	**Comorbidity**	**Associated symptoms**	**Drug history**	**Laboratory findings**	**Severity/outcome**
Locatelli, A. G. (40)	–	Dysgeusia, mild diarrhea	–	–	Non-severe
Alramthan, A. (29)	–	Asymptomatic	–	–	–
Suarez-Valle A. (36)	–	–	–	D-dimer↑, fibrinogen↑	Non-severe
de Masson, A. (44)	–	–	–	–	–
Freeman EE. (45)	HTN (2), obstructive lung disease (2), reumatologic disease (2)	Fever (9), cough (9), dyspnea (6), sore throat (5), headache (7), malaise (4), asymptomatic (5)	–	–	Outpatient care only (18), Hospitalized (5), Death (2)
Le Cleach L. (54)	Raynaud syndrome (2)	Fever (2), cough (2), dyspnea (3), asthenia (5), myalgia (3), headache (7), odynophagia (3), anosmia/ageusia (5), asymptomatic (3)	–	–	Outpatient (10)
Najarian, D. J. (20)	–	Cough, pain in leg and hands	Azithromycin, Benzonatate	–	Non-severe
Sanchez, A. (23)	T2D, HTN, peripheral artery disease, chronic renal failure	Fatigue, fever, dyspnea, acute respiratory distress	Cefpodoxime	EBV PCR positive (reactivation of EBV)	Severe/ Death
Hunt, M. (12)	–	Fever	–	Lymphopenia, CRP↑	Severe
Ahouach, B. (25)	–	Fever, dry cough	Paracetamol	–	–
Avellana Moreno, R. (34)	–	Fever, myalgia, asthenia, cough, diarrhea	Paracetamol	–	–
Reymundo A. (47)	–	–	–	–	–
Morey-Olive, M. (19)	Cholestatic liver disease (1)	Low-grade fever	Oral symptomatic treatment	Worsening of the markers for cholestasis	–
Gianotti, R. (22)	–	Fever, sore throat, cough	Levofloxacin (3), HCQ (3)	–	Mild (2), Moderate (2), Severe (1)
Mahé, A. (16)	T2D	Fever, asthenia, cough	Paracetamol	–	Non-severe/Survived
Robustelli Test, E. (42)	–	–	Lopinavir/ritonavir, HCQ (3 weeks before)	–	–
Zengarini, C. (28)	Moderate obesity, a history of alcoholism, and various chronic morbidities	Progressive dyspnoea, fever	HCQ, Omeprazole, Piperacillin/Tazobactam, Remdesevir, Potassium canreonate, and Enoxaparine.	–	Severe
Amatore, F. (35)	–	Fever	HCQ	Blood count: NL, electrolytes: NL, CRP: NL, anti-DNA antibodies: NL	
Gargiulo L. (48)	–	Fever	Paracetamol, Darunavir/cobicistat, HCQ	–	Severe, Death
Ciccarese G. (50)	–	Fever, sore throat, fatigue, hyposmia	Cefixime (3 days earlier discontinued), IVIG, Methylprednisolone	Leukocytosis, Lymphocytosis, severe Thrombocytopenia, LFT↑, LDH↑	–
Recalcati S. (6)	–	–	–	–	–
Gianotti, R. (21)	–	Fever (2), cough (2), headache (1), arthralgias (1)	Lopinavir/Ritonavir (1), Heparin (1), Levofloxacin (2), Ceftriaxone (1), Azithromycin (1), HCQ (1)	CRP↑, fibrinogen↑, ALT↑, AST↑	Mild (1), Severe (2)
Jimenez-Cauhe J. (41)	–	–	Lopinavir/Ritonavir, HCQ, Azithromycin, Corticosteroids, Ceftriaxone	Laboratory tests at the time of skin lesions showed worsening of one or more parameters compared to those at the time of discharge (CRP↑, D-dimer↑, lymphocyte count↓)	–
Galván Casas C. (24)	–	Cough, dyspnea, fever, asthenia, headache, nausea/vomiting/diarrhea, anosmia, ageusia, pneumonia	–	–	*Pseudo-chilblain:* less severe *Vesicular lesions:* intermedium severity *Urticarial and maculopapular lesions:* more severe COVID-19 disease *Livedoid/necrotic lesions:* the most severe disease
Estébanez, A. (5)	–	Dry cough, nasal congestion, fatigue, myalgias, arthralgias, diarrhea, ageusia, anosmia	Paracetamol	–	–
Gunawan, C. (43)	HTN, diabetes, dyslipidemia, hyperuricemia	Fever, cough, dyspnea, diarrhea	Azithromycin, HCQ, Cefoperazone-sulbactam, Omeprazole, medicines for his comorbidities	–	Non-severe
Quintana-Castanedo, L. (26)	–	4-day history of progressive cutaneous rash	–	–	–
Henry, D. (30)	–	Odynophagia, diffuse arthralgia, chills, chest pain, fever	Paracetamol	Moderate lymphopenia, CRP↑	Non-severe
van Damme, C. (33)	Obesity, T1D, hypercholesterolemia, HTN, obstructive sleep apnea-hypopnea syndrome, stroke 18 months ago without further sequelae, kidney failure on dialysis	General weakness, fever	–	Mild lymphopenia, CRP↑, LFT (GOT, GPT, LDH, GGT)↑	Severe/Death
Paolino, G. (38)	–	7th postpartum day, fever, dry cough, myalgia, arthralgia	Paracetamol	–	–
Proietti I. (55)	–	Asymptomatic	–	Normal	–
Zulfiqar, A. A. (17)	HTN, autoimmune hypothyroidism	Fatigue, fever, dry cough, abdominal discomfort, epistaxis	IV Amoxicillin–Clavulanic acid, LMWH	CRP↑, LFT showed cholestasis, progressive thrombocytopenia, fibrinogen↑, TPO↑	–
Joob, B. (13)	–	Fever, pneumonia, bleeding presentation (firstly missed diagnosed to be dengue)	–	–	–
Diaz-Guimaraens, B. (7)	HTN	Fever, pleuritic chest pain, shortness of breath	Telmizartan, HCQ, LR, Azithromycin	Lymphopenia, CRP↑, D-dimer↑	Non-severe
Magro, C. (18)	Obesity-associated sleep apnea, anabolic steroid use	Fever, cough, dyspnea, diarrhea, chest pain, myalgia	HCQ, Azithromycin, Remdesivir, prophylactic Enoxaparin	D-dimer↑, INR↑, CH50↑, C3↑, C4↑, Thrombocytopenia	Severe
Recalcati S. (6)	–	–	–	–	–
Zhang, Y (14)	HTN, DM, CAD	Fever, cough, dyspnea, diarrhea	LMWH	D-dimer↑, fibrinogen↑, FDP↑, PT↑	Severe/Death (5)
Bosch-Amate X. (46)	–	Fever, asthenia, cough, shortness of breath	HCQ, Azithromycin, LR, LMWH, Fondaparinux	Leukopenia, CRP↑, D-dimer↑	Hospitalized, Survival
Bouaziz, J. D. (39)	–	–	–	–	–
Marzano, A. V. (27)	–	Fever (21), cough (16), headache (11), weakness (11), coryza (10), dyspnea (9), hyposmia (4), hypogeusia (4), pharyngodynia (1), diarrhea (1), myalgia (1)	–	–	Mild (10), Moderate (2), Severe (10)/ Death (3)
Fernandez-Nieto, D. (37)	Atopic dermatitis (5), chronic urticaria (2)	–	7 patients: Lopinavir/Ritonavir (5), HCQ (6), Azithromycin (2)	–	Mild (14), Moderate (9), Severe (1)
Tammaro A. (32)	–	–	–	–	–
Potekaev NN. (53)	–	Fever (2), cough (1), weakness (1), shortness of breath (1)	HCQ (1)	–	Severe (2)
Freeman EE. (56)	HTN (32), diabetes (19), obstructive lung disease (14), Non-obstructive lung disease (9), cardiovascular disease (5), kidney disease (5), reumatologic disease (5), hidradenitis suppurativa (2), contact dermatitis (5), alopecia areata (4), melanoma (3)	Fever (103), cough (92), dyspnea (64), sore throat (62), headache (54), diarrhea, vomiting or nausea (51), malaise (45), myalgia (35), irratibility/confusion (27), chest pain (27), abdominal pain (23), anosmia (18), dysgeusia (12), arthralgia (16), rhinorrhea (14), asymptomatic (11)	Bevacizumab (12), Remdesivir (9), Lopinavir/Ritonavir (2), supportive care only (96), anti-malarials (41), antibiotics (40), serpin inhibitors (6), IL-6 inhibitors (4), JAK inhibitors (2)	–	Out-patient (95), Hospitalized (17), Non-invasive ventilation or high flow oxygen, ventilator and/or ECMO required (24), Death (8)
Matar S. (51)	–	–	–	–	–
Ho WYB. (52)	–	–	Lopinavir/Ritonavir	–	–

**Table 4:** Summary of characteristics of the patients based on the type of lesions.

**Characteristics**	**Chilblain-like lesions**	**Vesicular lesions**	**Urticaria-like lesions**	**Maculopapular lesions**	**Vascular lesions**	**Miscellaneous**	**Total**
*N* (%)	110 (18.4)	89 (15)	89 (15)	223 (37.3)	55 (9.2)	31 (5.2)	597
**Sex**, N^*^	97	83	85	219	46	11	541
Male, *n* (%)	43 (44)	42 (51)	28 (33)	107 (49)	28 (61)	2 (18)	250 (46)
Female, *n* (%)	54 (56)	41 (49)	57 (67)	112 (51)	18 (39)	9 (82)	291 (54)
**Age, mean**	40.7	56.1	46.3	56.4	72.3	48	53.3
**Rash location**							
Trunk, *n*	0	85	91	143	21	23	363
Upper Limb, *n*	24	44	29	194	8	9	308
Lower Limb, *n*	53	42	30	188	18	10	341
Head/Neck, *n*	0	12	20	42	0	7	81
Palms/Soles, *n*	1	2	2	1	1	1	8
Acral area (Finger, Toe), *n*	69	0	1	0	18	0	88
The mucous membrane, *n*	0	2	1	4	4	2	13
**Associated cutaneous symptoms**, ***n*** **(%)**	74 (67)	62 (70)	80 (90)	159 (71)	18 (33)	4 (13)	397 (66)
Pruritus, *n* (%)	28 (38)	55 (89)	22 (27.5)	126 (79)	4 (22)	3 (75)	238 (60)
Burning, *n* (%)	40 (54)	11 (18)	7 (9)	23 (14)	2 (11)	0 (0)	83 (21)
Pain, *n* (%)	47 (63.5)	9 (14.5)	6 (7.5)	19 (27)	3 (17)	0 (0)	84 (21)
**The onset of the lesions in relation toother symptoms**, ***N***	91	86	85	222	41	17	542
Before, *n* (%)	10 (11)	5 (6)	5 (6)	14 (6)	1 (2)	1 (6)	36 (7)
Chief complaint, *n* (%)	13 (14)	0 (0)	3 (3.5)	3 (1)	0 (0)	0 (0)	19 (3.5)
At the same time, *n* (%)	21 (23)	17 (20)	40 (47)	92 (41)	16 (39)	1 (6)	187 (34.5)
After, *n* (%)	45 (49)	64 (74)	37 (43.5)	113 (51)	24 (58.5)	15 (88)	298 (55)
**Median duration of skin lesions, day**	14	9	5.25	7.4	9.5	9.3	9
**Rash treatment**, N^**^	0	0	7	15	5	7	34
Without treatment, *n* (%)	0 (0)	0 (0)	0 (0)	7 (47)	1 (20)	0 (0)	8 (23.5)
Antihistamines, *n* (%)	0 (0)	0 (0)	4 (57)	0 (0)	1 (20)	0 (0)	5 (15)
Topical corticosteroids, *n* (%)	0 (0)	0 (0)	1 (14)	1 (7)	2 (40)	1 (14)	5 (15)
Systemic corticosteroids, *n* (%)	0 (0)	0 (0)	2 (28.5)	7 (47)	2 (40)	5 (71)	16 (47)
Hydroxychloroquine, *n* (%)	0 (0)	0 (0)	0 (0)	0 (0)	0 (0)	1 (14)	1 (3)
**Comrbidity**, N^***^ **(%)**	20 (19)	14 (13)	13 (12)	43 (40)	15 (14)	2 (2)	107 (100)
Hypertension	6 (30)	3 (21)	5 (38)	16 (37)	12 (80)	0 (0)	42 (39)
Diabetes	0 (0)	2 (14)	6 (46)	10(23)	6 (40)	1 (50)	25 (23)
Previous dermatologic illness^****^	2 (10)	8 (57)	1 (8)	10 (23)	0 (0)	0 (0)	21(20)
Obstructive lung disease	2 (10)	1 (7)	1 (8)	6 (14)	6 (40)	0 (0)	16 (15)
Non-obstructive lung disease	1 (5)	1 (7)	3 (23)	4 (9)	0 (0)	0 (0)	9 (8)
Rheumatologic disease	6 (30)	1 (7)	0 (0)	1 (2)	1 (7)	0 (0)	9 (8)
Chronic kidney disease	0 (0)	0 (0)	1 (8)	6 (14)	0 (0)	0 (0)	7 (6.5)
Cardiovascular disease	1 (5)	0 (0)	0 (0)	3 (7)	2 (13)	0 (0)	6 (6)
Obesity	0 (0)	0 (0)	1 (8)	0 (0)	1 (7)	1 (50)	3 (3)
Obstructive sleep apnea	0 (0)	0 (0)	1 (8)	0 (0)	1 (7)	0 (0)	2 (2)
Dyslipidemia	0 (0)	0 (0)	2 (15)	0 (0)	0 (0)	0 (0)	2 (2)
Liver disease	0 (0)	0 (0)	0 (0)	1 (2)	0 (0)	0 (0)	1 (1)
Peripheral artery disease	0 (0)	0 (0)	0 (0)	1 (2)	0 (0)	0 (0)	1 (1)
Autoimmune hypothyroidism	0 (0)	0 (0)	0 (0)	0 (0)	1 (7)	0 (0)	1 (1)
Hyperuricemia	0 (0)	0 (0)	1 (8)	0 (0)	0 (0)	0 (0)	1 (1)
Stroke	0 (0)	0 (0)	1 (8)	0 (0)	0 (0)	0 (0)	1 (1)
Alcoholism	0 (0)	0 (0)	0 (0)	0 (0)	0 (0)	1 (50)	1 (1)
**Associated symptoms**, N^*****^	96	58	83	210	43	6	496
Fever, *n* (%)	38 (39.5)	48 (83)	63 (76)	169 (80)	36 (84)	5 (83)	359 (72)
Cough, *n* (%)	35 (36)	40 (69)	51 (61)	146 (69.5)	31 (72)	1 (17)	304 (61)
Fatigue/Myalgia, *n* (%)	42 (44)	33 (57)	46 (55)	113 (54)	15 (35)	2 (33)	251 (51)
Dyspnea, *n* (%)	27 (28)	22 (38)	33 (40)	120 (57)	27 (63)	1 (17)	230 (46)
Headache, *n* (%)	32 (33)	24 (41)	26 (31)	64 (30)	7 (16)	0 (0)	153 (31)
Nausea/Vomiting/Diarrhea/Abdominal discomfort, *n* (%)	17 (18)	14 (24)	31 (37)	77 (37)	14 (32.5)	0 (0)	153 (31)
Dysosmia/Dysgeusia, *n* (%)	21 (22)	16 (27.5)	22 (26.5)	39 (18.5)	3 (7)	1 (17)	102 (20.5)
Sore throat, *n* (%)	13 (13.5)	10 (17)	11 (13)	33 (16)	3 (7)	1 (17)	71 (14)
Chest pain, *n* (%)	2 (2)	4 (7)	8 (10)	12 (6)	2 (5)	0 (0)	28 (6)
Nasal congestion/Coryza, *n* (%)	5 (5)	11 (19)	5 (6)	4 (2)	0 (0)	0 (0)	25 (5)
Irritability/Confusion	3 (3)	6 (10)	5 (6)	13 (6)	0 (0)	0 (0)	27 (5)
Arthralgia, *n* (%)	1 (1)	3 (5)	6 (7)	10 (5)	1 (2)	0 (0)	21 (4)
Bleeding presentation, *n* (%)	0 (0)	0 (0)	0 (0)	0 (0)	2 (5)	0 (0)	2 (0.4)
Chills, *n* (%)	0 (0)	0 (0)	1 (1)	0 (0)	0 (0)	0 (0)	1 (0.2)
Odynophagia, *n* (%)	3 (3)	0 (0)	1 (1)	0 (0)	0 (0)	0 (0)	4 (0.8)
Asymptomatic, *n* (%)	16 (17)	0 (0)	4 (5)	3 (1)	0 (0)	0 (0)	23 (5)
**Laboratory Findings**, N^******^	3	0	2	10	26	12	53
D-dimer increase, *n* (%)	3 (100)	0 (0)	0 (0)	0 (0)	12 (46)	3 (25)	18 (34)
Fibrinogen increase, *n* (%)	2 (67)	0 (0)	0 (0)	0 (0)	8 (31)	0 (0)	10 (19)
FDP increase, *n* (%)	0 (0)	0 (0)	0 (0)	0 (0)	7 (27)	0 (0)	7 (13)
PT/INR increase, *n* (%)	0 (0)	0 (0)	0 (0)	0 (0)	6 (23)	0 (0)	6 (11)
CRP increase, *n* (%)	0 (0)	0 (0)	1 (50)	3 (30)	3 (11.5)	2 (17)	9 (17)
Leukopenia, *n* (%)	0 (0)	0 (0)	0 (0)	0 (0)	1 (4)	0 (0)	1 (2)
Leukocytosis, *n* (%)	0 (0)	0 (0)	0 (0)	0 (0)	0 (0)	1 (8)	1 (2)
Lymphopenia, *n* (%)	0 (0)	0 (0)	1 (50)	2 (20)	1 (4)	2 (17)	6 (11)
Thrombocytopenia, *n* (%)	0 (0)	0 (0)	0 (0)	0 (0)	2 (8)	1 (8)	3 (6)
LFT increase, *n* (%)	0 (0)	0 (0)	0 (0)	3 (30)	1 (4)	1 (8)	5 (9)
CH50, C3, C4increase, *n* (%)	0 (0)	0 (0)	0 (0)	0 (0)	1 (4)	0 (0)	1 (2)
**COVID-19 treatment**, ***n*** **(%)**	46	40	69	185	39	10	389
Paracetamol, symptomatic or without treatment, *n* (%)	33 (72)	21 (52.5)	39 (56.5)	103 (56)	5 (13)	3 (30)	204 (52)
Chloroquine/Hydroxychloroquine, *n* (%)	9 (19.5)	16 (40)	27 (39)	90 (49)	26 (67)	8 (80)	176 (45)
Lopinavir/Ritonavir, *n* (%)	3 (6.5)	7 (17.5)	14 (20)	52 (28)	9 (23)	5 (50)	90 (23)
Azithromycin, *n* (%)	2 (4)	7 (17.5)	13 (20)	38 (20.5)	4 (10)	4 (40)	68 (17)
Other antibiotics^*******^, *n* (%)	4 (9)	4 (10)	7 (10)	22 (12)	12 (31)	4 (40)	53 (14)
Systemic corticosteroids, *n* (%)	1 (2)	3 (7.5)	5 (7)	19 (10)	5 (13)	4 (40)	37 (9.5)
NSAIDs, *n* (%)	4 (9)	1 (2.5)	3 (4)	10 (5)	17 (43.5)	0 (0)	35 (9)
Tocilizumab (IL-6 inhibitors), *n* (%)	2 (4)	2 (5)	4 (6)	9 (5)	5 (13)	0 (0)	22 (6)
Bevacizumab, *n* (%)	0 (0)	2 (5)	4 (6)	6 (3)	0 (0)	0 (0)	12 (3)
LMWH, *n* (%)	0 (0)	0 (0)	0 (0)	1 (0.5)	9 (23)	1 (10)	11 (3)
Remdesivir, *n* (%)	1 (2)	1 (2.5)	2 (3)	4 (2)	2 (5)	1 (10)	11 (3)
Serpin inhibitors, *n* (%)	1 (2)	1 (2.5)	1 (1)	3 (2)	0 (0)	0 (0)	6 (1.5)
Omeprazole, *n* (%)	0 (0)	0 (0)	1 (1)	0 (0)	0 (0)	1 (10)	2 (0.5)
JAK inhibitors, *n* (%)	2 (4)	0 (0)	0 (0)	0 (0)	0 (0)	0 (0)	2 (0.5)
Telmizartan, *n* (%)	0 (0)	0 (0)	0 (0)	0 (0)	1 (2.5)	0 (0)	1 (0.2)
Fondaparinux, *n* (%)	0 (0)	0 (0)	0 (0)	0 (0)	1 (2.5)	0 (0)	1 (0.2)
Darunavir/cobicistat, *n* (%)	0 (0)	0 (0)	0 (0)	0 (0)	0 (0)	1 (10)	1 (0.2)
IVIG, *n* (%)	0 (0)	0 (0)	0 (0)	0 (0)	0 (0)	1 (10)	1 (0.2)
COVID-19 severity^********^, ***N***	96	63	79	201	44	4	487
Mild, *n* (%)	79 (82)	32 (51)	40 (51)	79 (39)	2 (5)	2 (50)	234 (48)
Moderate, *n* (%)	13 (14)	18 (29)	28 (35)	86 (43)	12 (27)	0 (0)	157 (32)
Severe, *n* (%)	4 (4)	13 (21)	11 (14)	36 (18)	30 (68)	2 (50)	96 (20)
**Death**, ***n*** (%)^*********^	4 (3.6)	3 (3.4)	2 (2.2)	7 (3.1)	10 (18.2)	1 (3.2)	27 (4.5)

*FDP, fibrinogen degradation product; PT, prothrombin time; LFT, liver function test*.

* *Number of patients that their gender is reported in the articles*.

** *Number of patients that articles mentioned their specific treatment for the lesions*.

*** *Number of patients reported having comorbidities in the articles*.

**** *E.g., atopic dermatitis, chronic urticaria, melanoma, alopecia areata, hidradenitis suppurativa*.

***** *Number of patients that articles mentioned their associated symptoms*.

****** *Number of patients that their laboratory findings are reported in the articles*.

******* *Including mainly Levofloxacin, Amoxicillin–clavulanic acid, Cefpodoxime, Ceftriaxone, Piperacillin/tazobactam, Cefoperazone-sulbactam*.

******** *Mild: outpatients, Moderate: hospitalized patients (with or without supplemental oxygen), Severe: ICU added patients, non-invasive/invasive ventilation or ECMO required, patients with acute respiratory distress syndrome (ARDS)*.

********* *N is the total number of cases in each category; Chilblain-like lesions (110), Vesicular lesions (89), Urticaria-like lesions (89), Maculopapular lesions (223), Vascular lesions (55), Miscellaneous (31), Total (597)*.

Elevated D-dimer was the main laboratory finding in most of the cases, especially in patients with chilblain-like (100%) and vascular (46%) lesions. Disruption of coagulation condition (increase in PT, INR, and fibrinogen) was reported in patients with chilblain-like and vascular lesions (Tables 3, 4).

Regarding the drug history and medication regimen used for COVID-19, data of 389 out of 597 cases were available, most of which related to maculopapular and urticaria-like lesions. Fifty-two percentage of all cases and 72% of cases with chilblain-like lesions underwent symptomatic treatment with paracetamol, etc., or recovered without any medication. Chloroquine/hydroxychloroquine was the most common medication used in patients (45%). Details in Tables 3, 4.

Most patients had mild disease (48%). The majority of patients with chilblain-like lesions had mild disease (82%) and the majority of patients with vascular lesions had severe disease (68%). Also, most of the patients with maculopapular lesions were moderate (43%) regarding severity (Tables 3, 4).

The overall mortality rate among COVID-19 patients with cutaneous manifestations was 4.5%. Patients with vascular lesions had the highest mortality rate (18.2%) and patients with urticaria-like lesions had the lowest mortality rate (2.2%).

Details indicating characteristics of the lesions and the patients are shown in Tables 2–4.

## Discussion

After 1 year from the beginning of COVID-19 pandemic, the world is still facing a crisis. According to the current literature, more than half of the patients are asymptomatic leading to uncontrolled transmission of the virus (57–60). Recognizing COVID-19 related cutaneous manifestations may assist clinicians in early diagnosis of disease, before the development of respiratory symptoms, and may also be used to identify complications requiring treatment. The current study found that 10.5% of the COVID-19 patients reported skin lesions before the initiation of other symptoms or as their chief complaint. On the other hand, considering cutaneous manifestations is important to make the right diagnosis; as Joob et al. reported a COVID-19 patient with petechiae misdiagnosed with dengue fever (13). Our data demonstrated that 34.5% of cutaneous manifestations occurred at the same time with other symptoms particularly urticaria-like lesions (47%). It may suggest that urticaria-like lesions may be a diagnostic sign for COVID-19. The rest of the skin manifestations appeared later in the course of the disease and mainly after the initiation of systemic symptoms (55%) in our review. Galván Casas et al. suggested the chilblain-like and vesicular lesions as epidemiological markers for the disease (24). However, in our study, vesicular lesions (74%) were the most important cutaneous manifestations usually appearing after systemic symptoms of the disease.

Most of the patients with skin manifestations were middle-aged females, while, patients with chilblain-like lesions were younger (mean age: 40.7 years) and patients with vascular lesions were older individuals (mean age: 72.3 years). These findings are along with other studies about the chilblain-like lesions (6, 19, 24, 40). Maculopapular lesions were the most common dermatologic presentation of COVID-19 patients that commonly appeared at extremities. It occurred most often in middle-aged patients and was associated with moderate COVID-19 severity.

The overall mortality rate between the COVID-19 patients with skin presentations was 4.5%, with the point that there was the lowest mortality rate among the patients with urticaria-like lesions (2.2%) and contradictory, there was the highest mortality rate among the patients with vascular lesions (18.2%). Previous studies showed a pooled mortality rate of 3.2–6% in patients with COVID-19 (61, 62). Thus, the mortality rate of COVID-19 patients with skin manifestations is proportionate to the overall mortality rate of the disease.

Regardless of the type of skin lesions, 80% of COVID-19 patients with cutaneous manifestations experienced a mild and moderate, and 20% a severe COVID-19 disease. A previous study from the Chinese Center for Disease Control and Prevention reported that 81% of COVID-19 patients had a mild, 14% a severe, and 5% a critical disease (63). We don't have any specific data on patients without skin manifestations but comparing the COVID-19 severity in patients with skin manifestations and COVID-19 patients, regardless of their symptoms, demonstrates no obvious difference. Future cohort studies are required to compare the disease severity and outcome of COVID-19 patients with and without skin manifestations.

There is a wide range of cutaneous manifestations related to COVID-19 that in terms of age, associated symptoms, comorbidity, medication, severity, and mortality, chilblain-like lesions, and vascular lesions are the ends of this spectrum. Chilblain-like, urticaria-like, vesicular, maculopapular, miscellaneous, and vascular lesions are associated with an increase in COVID-19 severity and worsening the prognosis, respectively. Vascular lesions were more prevalent in males (61%) compared to females (39%). Considering the more severe disease and higher mortality rate in patients with vascular lesions, we can conclude that COVID-19 is more severe in males compared to females. This finding is compatible with our recent article, in which we assessed the sex-specific risk of mortality in COVID-19 patients (62).

Up to date, there is conflicting information about the potential possibility of transmitting the virus through the skin (37, 40, 64). Further investigations are required to identify the pathophysiology of SARS-COV-2 and to determine whether patients with long-lasting skin lesions (e.g., chilblain-like lesions) are capable of infecting other individuals through skin contact or not.

The overall frequency of cutaneous manifestations in COVID-19 patients was 5.95%, with a range from 0.2% up to 20.4% in different studies (6, 65).

Although skin presentations of COVID-19 are well described, the pathogenesis of skin lesions remains unknown. The direct viral invasion of the skin cells may be one possibility. Angiotensin-converting enzyme 2 (ACE2) is known as a ligand for the Spike protein of SARS-CoV-2 for entering human cells (66). There is a high expression of ACE2 on keratinocytes and sweat gland cells, respectively (67, 68). Thus, SARS-CoV-2 can directly infect keratinocytes resulting in necrosis. This hypothesis is consistent with our histologic findings which demonstrated the epidermal and adnexal necrosis in all skin lesions except vesicular rashes. According to Amatore et al., neither viral-induced cytopathic alterations nor intranuclear inclusions were seen in skin biopsies (35). However, SARS-CoV-2 spike and envelope proteins were detected in the endothelial cells of damaged skin in two cases with purpuric rashes (22). RT-PCR for SARS-CoV-2 was performed on skin samples of some patients and was negative in all of them. Since the nasopharyngeal swabs of these patients were positive simultaneously, we assume that it can be a false negative result due to a small viral load or technical problems. Further research is urgently needed.

Skin lesions during SARS-CoV2 infection might be immune-related phenomena. It has been shown that the presence of virus RNA in blood is related to greater severity of infection (69). Viremia is also associated with the levels of cytokines and growth factors in a dose-dependent manner with markedly higher levels in patients suffering from more severe COVID-19 (69). Recognition of the viral RNA by Toll-free receptors like TLR7 stimulates the intracellular signaling pathways which in turn enhance the cytokine secretion (69).

In a group of patients, with the end of the first week of the infection, a sharp increase in inflammatory cytokines such as interleukin (IL)1, IL2, IL7, IL10, granulocyte colony-stimulating factor (G-CSF), tumor necrosis factor (TNF) α and interferon (IFN)-g occurs. Overactivation of immune responses followed by pro-inflammatory cytokines increase may result in a “cytokine storm” which is an immune pathological condition (69–71). Increased cytokines allow them to access the skin, where they stimulate various cells, including lymphocytes, dendritic cells, macrophages, neutrophils, monocytes, and Langerhans cells to cause various skin manifestations (22, 69). Maybe a hyperviremia state is responsible for vascular lesions in severe COVID-19 patients. We suggest further investigations on the viral load levels among patients with vascular lesions compared with other skin manifestations.

The antigen-antibody complex can lead to complement activation and subsequent mast cell degranulation. This mechanism is suggested particularly for the urticaria-like lesions (43).

A low or delayed interferon response may result in uncontrolled viral replication followed by a subsequent cytokine storm which can lead to severe disease (72). Activation of the host immune system in response to viral antigen deposition may result in vascular damage in COVID-19 infection (73). It seems that high levels of type 1 interferon response, a critical factor in immunity against viral agents, is associated with chilblain-like lesions and mild disease (15, 72, 74). Activation and aggregation of cytotoxic CD8+ T cells and B cells also lead to lymphocytic thrombophilic arteritis and destruction of keratinocytes (21, 22). Nests of Langerhans cells are seen in most of the COVID-19 skin lesion biopsies and have been also reported in another viral-induced skin dermatitis-like pytriasis rosea (75).

Coinfection with other viruses is another potential possibility for COVID-19 related cutaneous manifestations. Some skin lesions in COVID-19 patients are very similar to rashes induced by other viruses like parvovirus18, herpes simplex virus type 1 and 2 (HSV-1, HSV-2), varicella-zoster virus (VZV), and poxviruses, both clinically and histologically. It is probable that because of the attenuation of the immune system, COVID-19 patients are susceptible to coinfection with or relapse of the other viral exanthems. This hypothesis is strongly suggested for vesicular and some miscellaneous lesions (e.g., erythema multiform) due to their unique histologic findings compared to other skin lesions of COVID-19 (24, 32, 37). A study reported four COVID-19 patients presenting diffuse vesicular lesions which microbiological and serological investigations demonstrated varicella infection (24). Thus, in COVID-19 patients with vesicular lesions, physicians need to investigate other possible etiological factors other than SARS-CoV-2.

Coagulopathy and vasculitis are other possible reasons for skin lesions during COVID-19. Evidence shows that COVID-19 patients are predisposed to coagulopathy and subsequent thrombotic events (76). It seems to be a result of inflammatory cytokine release, hypoxia, and other illness or therapeutic risk factors (76). Microvascular thrombosis of dermal vessels leads to ischemia or vasculitis mainly seen in chilblain-like or vascular lesions. Magro et al. focused on the role of the complement factors activation, especially alternative and lectin pathways, and subsequent thrombotic microvascular injuries (22). Evidence for this hypothesis is the elevated levels of CH50, C3, and C4 in blood samples as well as significant vascular depositions of C5b-9, C3d, and C4d in the dermis of skin specimens (22). According to our histologic findings mentioned in RESULT, vascular thrombosis was reported in almost all skin biopsies (except vesicular lesions). This finding across with the increased level of D-dimer, fibrinogen, and prolonged PT and INR in most patients is in favor of this hypothesis. Another presentation of coagulopathy in COVID-19 patients is hemorrhagic events and subsequent dermatologic manifestations (petechiae, purpura, and livedo). These manifestations are not specific to SARS-CoV-2. Schneider et al. reported a petechial rash associated with coronavirus NL63 (77, 78).

Extremely dilated blood vessels were introduced as a diagnostic histological finding for SARS-CoV-2 by Zengarini et al. (28). There are other reports of vasodilation and telangiectatic vessels in the dermis. With this finding, Magro et al. explained a possible pathway in which dysfunction of ACE2 (due to SARS-CoV-2 binding) and subsequent elevated level of angiotensin2 can result in high activation of endothelial nitric oxide synthase (eNOS) and ensuing vasodilation (22).

Drug-induced eruptions may occur during COVID-19. COVID-19 patients usually use a set of medications that potentially can cause cutaneous rashes. The current study found that paracetamol, azithromycin, hydroxychloroquine, lopinavir/ritonavir, and remdesivir were the most common medications used for COVID-19 patients. Paracetamol has been reported to cause asymmetrical drug-related intertriginous and flexural exanthema (STRIFE) (16). However, in Mahé et al. study, despite keeping the drug, skin lesions disappeared; that is very uncommon in drug reactions (16). Najarian et al. mentioned that maculopapular lesions of their patient could be according to azithromycin use or hypersensitivity reaction to azithromycin due to concurrent viral infection (20).

Hydroxychloroquine that has been used in 45% of all the cases (mentioned in Result) is one of the most likely medications to cause different skin rashes. Acute generalized exanthematous pustulosis (AGEP), erythroderma, urticaria, and erythema multiform are some of the skin lesions that have been reported in connection with hydroxychloroquine (79–81). However, Robustelli et al. mentioned that the skin lesion developed 3 weeks after discontinuation of the drug (42). As a conclusion, most of our reviewed articles considered the potential possibility of drug-induced exanthems but in almost all cases, dermatologic manifestations preceded the drug intake or the rashes disappeared despite the continuation of drugs (5, 7, 16, 20, 23, 37, 42, 43). So it is very unlikely that current COVID-19 medications are responsible for the reported skin lesions.

In our study, the prevalence of comorbidities in COVID-19 patients with skin manifestations is about 17.9% mainly reported in patients with maculopapular lesions. History of serious comorbidities like cardiovascular disease, hypertension, and obstructive lung disease was mostly reported in patients with vascular lesions; suggesting that patients with these skin manifestations are more complicated cases and need more attention. Interestingly, immune disorders were more common in patients with chilblain-like lesions. This finding is not reported yet and we suggest it to be focused on due to the possible relationship with the etiology and pathophysiology of these lesions.

Fever, cough, and dyspnea were more frequent in patients with vascular lesions and less frequent in patients with chilblain-like lesions. Also, 17% of patients with chilblain-like lesions were asymptomatic regarding systemic symptoms. Astonishingly, headache, dysosmia/dysgeusia, nasal congestion/coryza, and irritability/confusion were more common in patients with vesicular lesions. This finding can demonstrate the probable link between vesicular lesions and neurological manifestations. Future investigations are required to clarify the issue.

## Limitations

There were limited articles that mentioned complete data about all the items including the disease severity and outcome of the COVID-19 patients with dermatologic presentations. Another limitation was the absence of data about the COVID-19 patients without skin manifestations. Future cohort studies are required to compare the severity and prognosis of the disease in patients with and without skin manifestations, considering other related characteristics. Such studies help to better understand the prognostic value of the cutaneous manifestations in COVID-19 patients.

## Conclusions

Cutaneous lesions occur most often in middle age individuals at the same time or after the systemic symptoms of COVID-19. Urticaria-like lesions commonly (47%) occurred at the same time with other symptoms. It may suggest that urticaria-like lesions may be a diagnostic sign for COVID-19. A maculopapular rash is the main reported skin involvement in COVID-19 patients and is associated with intermediate severity of the disease. The mere occurrence of skin manifestations in COVID-19 patients is not an indicator for the disease severity, and it highly depends on the type of skin lesions. Chilblain-like and vascular lesions are the ends of a spectrum in which from chilblain-like to vascular lesions, the severity of the disease increases, and the patient's prognosis worsens. We highly suggest emergency and general practitioners to evaluate the suspected COVID-19 patients for any cutaneous manifestations. Those with vascular lesions should also be considered as high-priority patients for further medical care.

## Data Availability Statement

The original contributions presented in the study are included in the article/supplementary material, further inquiries can be directed to the corresponding author/s.

## Author Contributions

PJ, MN, and MM designed the study. PJ and BH performed the review literatures, collected the data, and wrote the first draft of the manuscript. PJ, HV, and MD helped in manuscript preparation. MM critically reviewed the manuscript.

## Conflict of Interest

The authors declare that the research was conducted in the absence of any commercial or financial relationships that could be construed as a potential conflict of interest.
